# The American Public Health Association

**Published:** 1882-01

**Authors:** 


### Societies.

### THE AMERICAN PUBLIC HEALTH ASSOCIATION.

The American Public Health Association convened in its ninth annual session at Savannah, Wednesday morning, November 29th, with the President, Dr. Charles B. White, of New Orleans, in the chair.

The exercises were opened with prayer by Rev. I. S. K. Axson.

Addresses of welcome were delivered by Mr. Geo. A. Mercer, in behalf the city, and by Dr. R. J. Nunn, in behalf of the Savannah Medical Society, which were followed by the annual message of the President.

The first business of the body was the election of a large number of new members.

The Secretary read a communication from the New

York Committee of the International Federation for the abolition of government regulation of prostitution. At its close, Hon. Erastus Brooks, of New York, announced that he had received copies of the *American Bulletin*, a paper published by the above named federation, which contained this communication, and which would be distributed among the members for their careful consideration. It was the earnest desire of the federation that this body co-operate with it in securing the abolition of all licensed prostitution, as it was a promoter of vice and accomplished no good purpose.

A resolution that the American Public Health Association obtain charters in the several States in which it had membership, was adopted; as were also resolutions to the effect that the Secretary of the Association be, *ex-officio*, a member of the Advisory Council, and that no two members from the same State can be, at the same time, members of such council.

Mr. Erastus Brooks then offered a resolution to the effect that the United States Congress be requested to take steps to secure co-operation between the Federal and various State governments to establish a uniform system of registration of births, deaths and marriages of the people of this country. Adopted.

The reading of papers being in order, Dr. Ezra W. Hunt, of New Jersey, read a paper on "The Contagious Diseases of Domestic Animals," in which he said: Foot and mouth disease has fortunately not planted itself on American soil. Contagious pleuro-pneumonia, although occasioning great loss of stock and of milk supply, has not as yet been proven to cause any specific disease through meat or milk to consumers, but it should be watched and studied by sanitarians. Hog cholera has prevailed in thirty States and has caused great mortality among swine. The subject of trichinosis received a general review. An ounce of hog's muscle has been found to contain 85,000 trichinæ. It was estimated that the body of a German, who died in a London hospital of the disease, contained 40,000,000 trichinæ and that the flesh of a hog that caused an epidemic in England contained 80,000,000.

Trichinæ have been found in all counties where search has been made for them. They are cosmopolitan with man, hog, rats and mice. As the disease does not pass rapidly from one animal to another there is much to encourage efforts for its eradication.

The New Orleans Sanitary Auxiliary Association submitted a report on "The Examination of Hogs at the New Orleans Abbattoir during the summer of 1881."

By this series of examinations it has been ascertained that Southern bred hogs are free from trichinæ. All the hogs infected with them are corn-fed animals. No mast-fed animal was found to be infected. The swill-slops and offal-fed hogs appeared to have the fairest and best meat when inspected by the unaided eye, but under the microscope their flesh presented a bad appearance, being cloudy and the cells often broken down and disorganized, as if the tissues were infiltrated with particles of dirt. The corn-fed hogs were the most solid and showed the best fibre. The mast-fed hogs were oily and soft. The clover-fed hogs were rather poor, and the fat had a yellowish tinge.

It was found to be a very easy matter to decide on what kind of food an animal had been raised, by an inspection after he had been slaughtered and dressed for market.

The sanitary recommendations were as follows:

An improved trough should be made to contain the food of the hogs, and corn should not be scattered over the floors of the pens, as is customary at present, for by this means the corn finds lodgment in the dung, and thereby becomes a vehicle of infection. The pens should be kept very clean. When one hog of a lot is found to be infected with trichinæ, the animals should be separated, and not more than one hog placed in a pen.

By a rigid system of inspections, it is possible to prevent the sale of fresh pork infected by the trichinæ spiralis.

Dr. S. S. Herrick, of New Orleans, presented a paper on "The Comparative Vital Movement of the White and Colored Races in the United States." It will be observed, he says, that the ratio of mortality for all ages is invariably much greater among the colored than among the white, and that the disparity is still more marked under five years of age.

It has long been known that the rate of mortality among the African race in this country is much greater than among the European, especially in the periods of infancy and early childhood; and it was generally supposed, until the census of 1880 corrected the mistake, that the former would gradually be extinguished in a state of freedom.

The count of 1880 showed a numerical increase of negroes over the census of 1870. [NOTE —May it not be that the returns were more complete, and included many persons omitted in the previous count? The relative mortality of negroes, in the cities at least, greatly exceeds that of the whites, and we doubt if the birth-rate fully compensates it.—EDS.]

A resolution was adopted to appoint a committee to examine the papers read on diseases in food animals, to report at the next meeting.

Dr. J. J. Speed, of Louisville, Ky., read a paper on the "Inside Sources of Disease." He took the ground that improper eating and excessive drinking produced more disease than marsh miasm and many other generally recognized causes of disease. He believed the prevention of disorders consequent upon inside causes came properly under the cognizance of the sanitarian and should receive his most careful attention.

Dr. A. J. Miles, of Ohio, was next announced, and proceeded to read a paper on "The History of Sunstroke Mortality in 1881," in the course of which he said: The foregoing observations, which have just been presented, establish this fact, that as far as Cincinnati is concerned, the thermometer being the same, a dry atmosphere is much more conducive to a sunstroke than a hot and moist atmosphere. The number of sunstrokes and prostration from heat in our city was truly alarming. Many persons who suffered from this prostration during the extreme weather were not exposed to the direct rays of the sun, but succumbed in some of the many factories or shops.

Many others were taken while employed at their daily avocations, and others while on their way to and from their places of business. Many of those who were overcome by the heat became unconscious at once, while others were simply prostrated; and again many more were taken at their houses and were not considered sunstruck.

No set line of treatment for sunstroke can be laid down. The treatment is entirely symptomatic, and needs careful and accurate observation, in order to be able to treat them scientifically or satisfactorily.

The symptoms demanding most attention and most active treatment were—

1. Temperature—(a) Elevated. (b) Depressed.

2. Heart—(a) Increased action being rare and not in any of our cases requiring treatment. (b) Depressed action calling forth all means, often without avail.

3. Convulsions—With opisthotonos, demanding careful administration of ether.

4 Respiration—(a) Difficult and slow, connected with spasmodic conditions of larynx. (b) Failure of respiration. Requiring artificial respiration; two patients were saved in this way.

After treatment, had reference to the condition left, the former habits, and the indications in the weather.

I. Prevention, ice caps and cold cloths to head.

II. Nourishing diet, tonics and small amounts of stimulants.

Method of treatment pursued.

I. Immediate treatment.

1. Temperature.

(a) Temperature below normal. Warm flannels, wrung out of hot water, and warm beef tea, by mouth or per rectum.

(b) Elevated Temperature. Were treated by cold in some form. Where 103 degrees and above, baths cooled from 95 degrees to 90 degrees, and careful watching of the temperature of the body to see that it did not get below normal. Where the case required repeated baths, sometimes cold packs and sprinkling with ice water was resorted to. Small pieces of ice, with the edges carefully rounded, were placed in the rectum until it was packed full, and in all cases ice caps were applied to the head, in or out of the bath.

2. Consciousness. In order to stimulate the return of consciousness, ice-cold water was poured on the forehead in small amounts, and in a continous stream from a height, thereby using the "reflex action" of the fifth nerve to arouse consciousness.

3. Convulsions were controlled by careful administration of ether, enough to get the cerebral centres quieted, yet not enough to produce depression in them.

4. Respirations were stimulated by ether and atropia hypodermically, using 15 M. ether, and 1-40 to 1-20 grain atropia.

5 Pulse and heart Failure of the heart in both "anæmic" and "congestive" varieties was a most alarming symptom. Intermittent action of the heart was controlled by digitalis, or by digitaline. The force of the heart was sometimes increased by it. The other agents used in stimulating the heart were whisky, ether, atropia and aqua ammoniæ.

6. In the marked cases of the congestive variety, hot water treatment was tried, baths of a temperature of 110, and patient immersed, with his head surrounded by ice caps, and with cold water poured on it, with the view of increasing the capillary circulation of the body, and thereby decreasingthe amount of blood going to the head. In all cases where this was tried it was unsuccessful.

Modes of administration and ways of reducing the temperature:

Reducing Temperature.-In all cases where the temperature was 102 deg. or below, sponging the naked body, first with tepid water and afterward with successively colder and colder water, advancing by a few degrees each time, seems the best, easiest and most satisfactory way.

Above 102 deg. baths cooled from 95 deg. to 90 deg. with careful watching, seemed the best, although circumstances required us to resort to "cold packs." A sheet closely tucked over the naked body, and using hydrant water at first, and in cases of persistent high temperature finally resorting to colder and colder water, until you reach ice water. In all cases, the heads were covered and surrounded by ice caps and bladders filled with ice.

As Regards Administration of Remedies.—The order to which preference should be given is about as follows, though we were guided by the effects obtained: First, by mouth; second, by rectum; third, by hypodermic; fourth, by inhalation.

By Mouth and Stomach.—In most severe cases unconsciousness, convulsions, vomiting, or other difficulties, made the administration by the mouth difficult and tedious, when rapidity was necessary, and in other cases where the stomach was overloaded with beer, etc., vomiting followed, so that so much time and medicine was lost. Again, in greatly prostrated cases, with very weak and rapid pulse, absorption, or the effect as noted from the medicine was slow in coming. Still, in certain cases where rapidity was not desired, and when it could not be used, it was found to be easy and undoubtedly proper, and in case the action is not sufficient, other means can be resorted to.

Rectum.—Many severe cases had relaxed sphincter and others had diarrhœa and involuntary discharge of fæces. In all cases of this kind the methods of administration by rectum had to be abandoned for others. Whisky, warm beef-tea, tincture of digitalis and carbonate of ammonia were used and the effect appeared to be better and more rapid than when administered by the mouth, and suitable to cases where, for other reasons, the stomach could not be reached.

By Hypodermic.—In cases where rapid action is necessary, and where other means fail, this can be used by combining a number of different agents. You can undoubtedly get a larger amount of stimulation than by any other means, and with much less danger of producing bad effects from too much of a single remedy, viz:

Digitaline for the heart when intermittent.

Ergotine for contracting the capillaries.

Atropia for stimulating heart and respiratory centres.

Whisky and aqua ammoniæ and ether for stimulating effect on the heart.

Carefully noting your case and its symptoms, you choose the one best adapted to your symptoms. If you do not get a good and rapid effect try the others; and when death seems inevitable, "heroic treatment," by hypodermic injections of aqua ammoniæ is justifiable and right. Experience seems to show us that deep injections of aqua ammoniæ, diluted one-fifth, do not often produce an abscess. You can use alcohol or water for diluting it.

Ether administered by inhalation produced three effects, not all noted in the same case.

1st. Sometimes stimulated pulse, and it became stronger and less frequent. 2d. Made respiration easier and deeper, and 3d, quieted convulsions.

The next paper was by Dr. J. F. Adams, of Massachusetts, on "Malaria in New England." The following points were considered:

1st. The periods when malaria has been present in New England.

2d. The nature of the localities visited.

3d. The rate of progress of the malarial wave.

4th. The subsidence of typhoid fever coincident with the appearance of malaria.

5th. The bearings of the facts observed upon the etiology of malaria.

The reading of this paper gave rise to an interesting discussion, which was opened by Dr. Henry F. Campbell, of Augusta, who took the ground that malaria had existed previous to its discovery as detailed in this paper, that it was so intimately blended with the typhoidal element in certain localities which were supposed to be free of malarial poison as to be scarcely distinguishable. His remarks were listened to with great attention, especially those in reference to the use of quinine in the treatment of malarial and typhoid malarial diseases.

Hon. Erastus Brooks, of New York, presented and read a paper on "The Duties of the Citizen to the State in Maintaining Public Health." He maintained that it was the duty of the State to protect the health and lives of citizens, and urged the necessity for the establishment and effective support of State and local boards of health. He insisted upon the importance of sanitary inspection of schools, public buildings and institutions, and expressed the conviction that many of the most fatal diseases that now curse the country could be stamped out of existence by strict observance of sanitary rules.

The report of Committee on Statistics, presented by Doctor Elisha Harris, of New York, was read by Doctor C. N. Hewitt, of Minnesota.

After the reading of the report, and the adoption of the resolutions embraced in it, the following general resolution was offered by the association and unanimously adopted.

"*Resolved*, That the Executive Committee of this association is hereby instructed to memorialize, in the name of this association, the Congress of the United States in favor of such legislation as will bring about a proper cooperation between the General Government of the United States and the several State Governments for a uniform and efficient system of the registry of the deaths, births and marriages of the population."

Dr. Gustavus Devron, of New Orleans, read a paper, prepared by Dr. C. B. White, Sanitary Director of the New Orleans Auxillary Association, on "The Yellow Fever Epidemic of 1878."

This valuable paper contained many instructive facts, and presented statistics of much interest connected with that fearful visitation.

"Impure Water and its Dangers" was the title of a paper by Dr. M. T. Runnels, of Indiana.

Dr. S. S. Herrick, of New Orleans, read a paper on "Railroad Sanitation," and Dr. B. Joy Jefferies, of Massachusetts, followed in an interesting address on "Color Blindness."

He referred to the examinations in Chatham Academy, where he found 22 color-blind boys among 274, a large per centage, the usual per cent. being four. Amongst 91 girls, none were found to be color blind. Of the Association members, two out of 73 tested were color blind, and none of the ladies. He advocated the education of boys in color names, by which their color sense would be improved, and thus gradually they will cease to be behind the girls in appreciation of color. Then the color blind boys will be detected in school, and they will not attempt to enter professions where our future laws must throw them out. He cited the instance of two color blind brothers, the sons of a Savannah pilot, who thus being warned, will not attempt to follow their father's calling, as the law now prohibits.

Within the past three years Dr. Jeffries has tested 19,001 males, of whom 801, or about 4 per cent were found to be color blind, and 14,633 females, of whom only 11 were color blind.

The committee on nominations made the following report, which was unanimously adopted:

President—Prof. R. C. Kedzie, of Michigan.

First Vice President—Dr. Ezra M. Hunt, of New Jersey.

Second Vice President—Dr. Albert L. Gihon, of United States Navy.

Treasurer—Dr. J. Berrien Lindsley, of Tennessee.

Executive Committee—Dr. James E. Reeves, of West Virginia; Dr. Stephen Smith, of New York; Dr. Thos. Neal of Ohio; Dr. J. G. Thomas, of Georgia; Edward Farmer, Esq., of Louisiana; Dr. John H. Rauch, of Illinois.

The Committee on Compulsory Vaccination and Revaccination submitted the following through their Chairman, Dr. James H. Letcher, of Kentucky.

*Resolved*, That this association, through its Committee on Compulsory Vaccination and Revaccination, ask all State medical societies, and State boards of health to endeavor as far as possible to procure the enactment of laws in their respective cities, counties and States, compelling vaccination and revaccination.

*Resolved*, That the American Public Health Association be asked to render its aid and influence in the furtherance of compulsory vaccination and revaccination.

*Resolved*, That the Governor of each State in the Union be communicated with concerning this matter, with the request that in the next annual message to the law makers, he call their attention to the importance of the speedy enactment of laws touching this question.

*Resolved*, That the United States Government, through its Chief Magistrate, have its attention directed to the need of a national law on compulsory vaccination and re-vaccination, and at all events that it be advised of the importance of compelling all foreigners who come to make their homes with us, unless they can give proof of successful vaccination, to be vaccinated immediately on arrival here.

A motion to lay the report on the table was made, but was subsequently withdrawn.

Dr. Ezra B. Hunt, of New Jersey, thought the subject one of great moment, and too important to be disposed of hastily, and opposed the summary motion of laying it on the table, but hoped that no action would be taken on it until next year.

Dr. H. B. Baker, of Michigan, moved to refer it to a special committee, when the secretary stated that this subject had already been considered by a special committee, and the resolutions was their report.

Dr. Hewitt, of Minnesota, objected to the resolutions, stating that previous to his leaving home, it was proposed to pass a compulsory law in that State, the Legislature then being in session, but he had protested against it. He thought there was a better remedy for the prevention of the disease than enacting compulsory laws. It was a great deal easier to persuade a man in an emergency than to drive him with a stick. The passage of such a law would result in trouble, and could accomplish no good. He saw no necessity for any action on the part of this association, and thought the matter could be better dealt with by the State Boards.

Dr. Hunt withdrew his motion, and seconded that of Dr. Baker to refer to a special committee.

The discussion was participated in by Dr. Campbell, of Augusta; Dr. Cochran, of Alabama; Dr. Chamberlain, of Connecticut, and Dr. Falligant, of Savannah, who did not favor compulsory laws, though strong advocates of general vaccination.

Dr. Devron, of Louisiana, said, if the adoption of the resolutions by the association carried them into immediate effect, he would be opposed to them, but it was not likely any decisive result on the subject would take place within the next year or two, possibly three. The effect, however, he thought would be good, if it went forth that this association had endorsed a scheme for stamping out malignant and contagious but preventable disease. [Applause.]

Dr. Thomas, of Savannah, was in favor of the most rigid sanitary laws on all subjects, but thought in this case they should make haste slowly. In this country, where personal liberty is so cherished, compulsory laws would inevitably arouse opposition, and might be productive of no good. He further doubted whether legally such a law could be passed.

Dr. A. M. Bell suggested that the matter be referred back to the same committee, and that the committee be enlarged

Dr. Baker withdrew his motion, and moved that the resolutions be recommitted to the same committee, with the request that they report back at the next meeting, giving their views and reasons for the recommendations, which motion finally prevailed.

By Medical Director, Albert L. Gihon:

*Resolved*, That it is the opinion of this association that the usefulness, powers and influence of the National Board of Health will be materially advanced by reconstituting it, as a representative body to be composed of representatives from the several State Boards of Health, the medical department of the United States, army and navy, and the United States Marine Hospital Service.

*Resolved*, That a committee of delegates, to be nominated by the several State Boards of Health, army, navy and Marine Hospital service, in the proportion of one from each, be instructed to consider, without delay, the best manner of making known to the Congress of the United States, the opinion of the Association to accomplish this result.

Dr. Gihon stated he had the warmest personal regard for the members of the National Board of Health, and that the resolution was offered in the belief that the National Board would expire at the end of the present fiscal year. It, however, appeared that the board was a permanent one, so long as Congress appropriated for it. The question was whether a change was desirable in the composition of the board. As one of the original members of the association, and an ardent and earnest worker for the establishment of a National Board, he thought he had a right to speak.

When the idea of a National Board was first created, many believed, and still believe, it should be a representative body. If it were more largely a representative board the chances are that it would be in a greater degree co-operative with and co-ordinate to the different State Boards of Health, and local prejudices would be removed.

Dr. Ames stated that we have had a National Board of Health for several years, and asked whether any one was willing to say it had not been a success, measurably—perfection of course could not be looked for. In order to accomplish the change, three things were necessary, viz: A bill through the House of Representatives, the Senate, and the signature of the President. To do this now would be almost impossible. Even if such a bill should pass, the body would be unwieldy, and the time would be taken up in useless discussion of individual views; and that to agitate a change now, so long before the expiration of the quarantine act, would be injurious both to the association and the National Board.

Dr. Reeves stated he thought the members mistook their position. The association was wholly voluntary. It bore no relation to State or Federal Governments, and it would be ample time to pass such resolutions when the State sent representatives here instructed to say the National Board should be re-organized.

Dr. Baker stated that, although it might not be desirable to pass the resolutions offered by Dr. Gihon, he thought a sanitary congress, composed, as suggested in the resolution, might be established, and might under certain circumstances sustain the National Board of Health.

Dr. Hunt offered the following resolution as a substitute:

*Resolved*, That it is the opinion of this association that the usefulness, power and influence of the National Board of Health will be materially advanced by no action on our part at present, and that the question of direct representation of State Boards of Health in it may safely be left to emanate from the State Boards themselves, instead of from a voluntary association.

In support of his resolution he stated that he agreed with what had been said relative to the injurious effect of passing Dr. Gihon's resolutions eighteen months before the expiratian of the quarantine act—injurious alike to the association and the National Board of Health. He also stated the association did not represent the whole country. The members present were not representatives of the State boards, and in a voluntary association of this character it does not behoove the association to take this action suggested by the original resolutions. To create such a board would be as absurd as to depose the Commissioner of Agriculture, Superintendent of the Census and others and substitute a representative board in their places. For his part if any change was desirable he thought it should look towards a reduction in size. Experience in England and other countries has established the fact that the smaller the body the more apt it is to be effective.

Dr. Devron stated if the matter were left to State boards nothing would be done. If the matter is brought before Congress at once, public interest will be awakened, and Representetives and Senators will be urged by their respective States to take action in the matter.

Dr. Baker offered the following as an amendment to Dr. Hunt's substitute:

*Resolved*, That this association deems it desirable that there should be a National Sanitary Congress, composed of one member from each State in the Union, members to be appointed as follows: In the States which have State Boards of Health by those boards, and in other States by Governors of States.

Dr. H. F. Campbell, of Augusta, said that any one who has watched with the deep and intense interest that he had the slow and halting progress of sanitation in the United States and especially in this his own State, must necessarily hesitate to disturb the condition of any attainment so valuable to the interests of American sanitation as the National Board of Health.

As early as 1847, when the convention to form the American Medical Association met in Baltimore, one of the first committees appointed was one on registration of vital statistics. At that time only Massachusetts, ever honorably in advance in sanitary matters, and New York, whose exact date he did not know, had boards of registration.

Now, at that very time, two States were in advance of all others in taking measures to follow the example of Massachusetts and New York. One was New Jersey, and the other, it may surprise many here to learn, was *Georgia*. But how slow has been the progress! It is true New Jersey went on, and has succeeded well in her Board of Health. But how has it been with Georgia? The early effort fell dead, and it was not until 1875 that, through a distinguished physician of Savannah, Dr. J. G. Thomas, did we succeed in securing an act of registration and a Board of Health. It was not, perhaps, a perfect act, but was attempted to be improved. It was tampered with, and the result was that our little pittance of fifteen hundred dollars was taken from us by our Legislature and our board has been inoperative to the present time.

This grand attainment of a National Board of Health is endangered by being tampered with. It may not be perfect, but it is a most valuable addition to the sanitary organization of the country. Its establishment is the culmination and the completion of that which has been the great object and desire of all the most earnest and thoughtful sanitarians of this country. It was only gained by persistent and noble efforts. Let us not endanger its existence by bringing it again before Congress. It will set political machinations to work, and we will find ourselves without this valuable and important element in American sanitation.

He claimed no priority in the matter, but he must record here, whatever is due to his colleague and friend, Dr. C. B. Nottingham, of Macon, in the suggestion of a National Board of Health. It was a prominent and earnestly advocated object of his life. He presented reports, made suggestions, and clearly formulated his plan. All he could do in behalf of its origination was faithfully done by this earnest man.

He has passed from among us; his voice is no more heard in our councils, or in moving the affairs of men. Though "he rests from his labors his works do follow him."

We have a National board of Health. It is acting in full concert with the State boards. You can influence the board to appoint its representatives in the State boards, but he would implore the association not to disturb the original act before Congress. There its life would be imperiled. It is too delicate and ticklish a matter to handle. Let us not endanger so valuable and all-important a possession.

Dr. Agnew stated the amendment seemed aimed at the existence of this association. The creation of a Sanitary Congress would perform the functions of the American Public Health Association, and its existence would be terminated.

The previous question was called, and the main question ordered.

The question first occurred on Dr. Baker's amendment, and it was lost.

The question next recurred on Dr. Hunt's substitute. On a division there were ayes 41, nays 18.

So Dr. Hunt's resolution was substituted for the original.

The question occurred on adoption of the substitute, and on a division there were ayes 41, nays 18, and it was adopted.

A paper by Dr. Stephen Smith, of New York, on "The Relations of State and Local Quarantine Systems to a National Quarantine System," was read by title.

Dr. W. C. Van Bibler, of Maryland, read a paper on "Two suggestions Concerning Healthful Buildings."

Papers by Dr. James L. Cabell. of Virginia, on "The Origin and Progress of International Hygiene;" by Mr. A. Y. P. Garnett, of the District of Columbia, on "Observations on the Potomac Marshes at Washington as a Pathogenic Agent in the Production of the So-called Malarial Fevers," and by Dr. Thomas J. Turner, U. S. N., "Some Remarks upon National and International Sanitary Jurisprudence," were by request of the authors, read by title.

The Association was the recipient of many courtesies, both public and private. The local committee was assiduous in its attentions. The attendance was large, and a keen interest was manifested in the proceedings. The association adjourned on the evening of the fourth day to meet in Indianapolis, Indiana, in October, 1882.

---

